# Correction and Republication: Symptoms of Depression, Anxiety, Post-Traumatic Stress Disorder, and Suicidal Ideation Among State, Tribal, Local, and Territorial Public Health Workers During the COVID-19 Pandemic — United States, March–April 2021

**DOI:** 10.15585/mmwr.mm7048a5

**Published:** 2021-12-03

On July 2, 2021, *MMWR* published “Symptoms of Depression, Anxiety, Post-Traumatic Stress Disorder, and Suicidal Ideation Among State, Tribal, Local, and Territorial Public Health Workers During the COVID-19 Pandemic — United States, March–April 2021” (*1*). On October 12, 2021, the authors informed *MMWR* that some data were inaccurate because 420 incomplete participant responses were incorrectly assigned scores for depression. This error resulted in a change in overall depression prevalence from 32.0% to 30.8%, and other similar changes in stratified prevalences of depression, prevalence ratios of depression, and the overall proportion of respondents who reported at least one mental health condition. The authors have corrected the *MMWR* report by excluding the 420 records from the depression analysis and confirmed that the interpretation and the conclusions of the original report were not affected by these corrections. *MMWR* has republished the report (*2*), which includes the original report with clearly marked corrections in supplementary materials.
